# Structure of the NheA Component of the Nhe Toxin from *Bacillus cereus*: Implications for Function

**DOI:** 10.1371/journal.pone.0074748

**Published:** 2013-09-10

**Authors:** Magdah Ganash, Danh Phung, Svetlana E. Sedelnikova, Toril Lindbäck, Per Einar Granum, Peter J. Artymiuk

**Affiliations:** 1 The Krebs Institute, Department of Molecular Biology and Biotechnology, University of Sheffield, Sheffield, United Kingdom; 2 Department of Food Safety and Infection Biology, Norwegian School of Veterinary Science, Oslo, Norway; SUNY Upstate Medical University, United States of America

## Abstract

The structure of NheA, a component of the *Bacillus cereus* Nhe tripartite toxin, has been solved at 2.05 Å resolution using selenomethionine multiple-wavelength anomalous dispersion (MAD). The structure shows it to have a fold that is similar to the *Bacillus cereus* Hbl-B and *E. coli* ClyA toxins, and it is therefore a member of the ClyA superfamily of α-helical pore forming toxins (α-PFTs), although its head domain is significantly enlarged compared with those of ClyA or Hbl-B. The hydrophobic β-hairpin structure that is a characteristic of these toxins is replaced by an amphipathic β-hairpin connected to the main structure via a β-latch that is reminiscent of a similar structure in the β-PFT *Staphylococcus aureus* α-hemolysin. Taken together these results suggest that, although it is a member of an archetypal α-PFT family of toxins, NheA may be capable of forming a β rather than an α pore.

## Introduction

*Bacillus cereus* is a well-known food poisoning organism, causing both emetic- and diarrheal type syndromes [1]. It can easily contaminate food production or processing systems. The diarrheal strains of *B. cereus* produce three enterotoxins, hemolysin BL (Hbl) [2,3] non-hemolytic enterotoxin (Nhe) [4], and cytotoxin K (CytK) [5]. While CytK is a single-component protein toxin [5,6], Nhe together with Hbl are tripartite enterotoxins. Of the three toxins, Nhe is probably the most important in diarrheal food poisoning, being presented in all food poisoning isolates [7]. The toxin was identified from a *B. cereus* strain lacking the genes encoding both CytK and Hbl, which was involved in a large food-poisoning outbreak in Norway in 1995 [4].

Nhe is a complex pore-forming toxin (PFT) consisting of three proteins, NheA (41-kDa), NheB (39-kDa), NheC (40-kDa), encoded by one operon containing three genes *nheA*, *nheB* and *nheC*, respectively [8]. Separately, these proteins show no toxicity, but as a binary complex some activities (NheA + NheB: ~5%) were seen and maximal activity is obtained only when all three components are presented (100%) [9]. The NheB and NheC components are able to bind to cell membranes [9,10], whereas NheA was believed to lack this ability, despite the fact that binding of NheA to NheB/NheC is thought to be the final stage of pore formation [10]. As a ternary complex in a molar ratio of 10:10:1 of NheA, NheB and NheC, respectively, the toxin reaches optimal cytotoxicity as demonstrated in Vero (monkey kidney epithelium) cell assays [9]. Patch clamp studies have shown that the combination of only NheA and NheB is sufficient to induce large-conductance channel insertions in non-epithelial cells, such as clonal rat pituitary cells (GH_4_ cells), but much smaller channels were observed in Vero cells [11]. "Nhe" was originally an abbreviation of “non-hemolytic enterotoxin” a name mistakenly given during the initial studies on the toxin. Subsequent investigations have, however, shown that the toxin is able to lyse erythrocytes from various organisms, including human, horse, cat, cow, dog and pig [12], although to a lesser extent than Hbl.

Several studies [9,10,11,12] have been conducted in order to understand the mechanism behind the pore-formation of the toxin but the detailed mechanism, and the detailed functional role of each of the Nhe proteins, remains unknown. The three Nhe proteins share sequence homology with one another and also with the three Hbl proteins [12]. By using the crystal structure of Hbl-B [13] as a template, Fagerlund et al. (2008) constructed homology models of NheB and NheC which suggested that they could adopt similar structures.

The crystal structure of Hbl-B [13] also revealed that it (and therefore by implication the other two Hbl proteins and all three Nhe proteins) has a marked structural resemblance to another toxin: the 34-kDa single-protein α-PFT cytolysin A (ClyA) of *Escherichia coli* [12,13,14]. In addition to the structural similarity, both Nhe and ClyA are cytolytic to epithelia and form large-conductance channels in planar lipid bilayers [12,15,16,17]. ClyA possesses a hydrophobic β-sheet region known as the "β-tongue" [14] which becomes part of a transmembrane (TM) α-helix as a result of the large-scale structural changes that accompany the insertion of ClyA into the membrane during pore formation [18]. The predicted β-tongue region in NheC is essential for binding to cell membranes, as the protein was not able to bind to cells when the entire hydrophobic stretch was removed [10], as had also been shown for ClyA [14]. Additionally, replacing the two cysteine residues located within the predicted β-tongue of NheC with glycines markedly impaired cytotoxicity [10], although the roles of these cysteines remain undefined in the protein. A specific monoclonal antibody against NheB neutralizes the toxic effect of the Nhe complex by preventing NheB from binding to NheC and NheA [19]. Unlike NheB and NheC, the region of the NheA sequence corresponding to the hydrophobic β-tongue in ClyA and Hbl-B is replaced by an amphipathic sequence, indicating that there can be expected to be major differences in functionality and possibly structure.

It is likely that pore-formation by the Nhe toxin follows a pattern involving membrane binding, oligomerization and finally insertion of the transmembrane regions to form the pore, as proposed in other pore forming toxins [20]. Crystal structures of the Nhe toxin components, whether in their soluble or pore forms, will be of great value in understanding the processes of pore formation, but until the present work no crystal structure of any part of the Nhe toxin has been available. We report here the crystal structure determination at 2.05 Å resolution of NheA by selenomethione Multiwavelength Anomalous Dispersion (MAD). The structure reveals a similar overall fold to *B. cereus* Hbl-B and *E. coli* ClyA, but, unlike them, its enlarged "head" domain displays on its surface an enlarged β-tongue which is of amphipathic rather than hydrophobic nature. The possible implications of this finding for the function of NheA in the mechanism of action of the toxin are discussed.

## Results

### Overall structure of NheA

**Figure 1:**
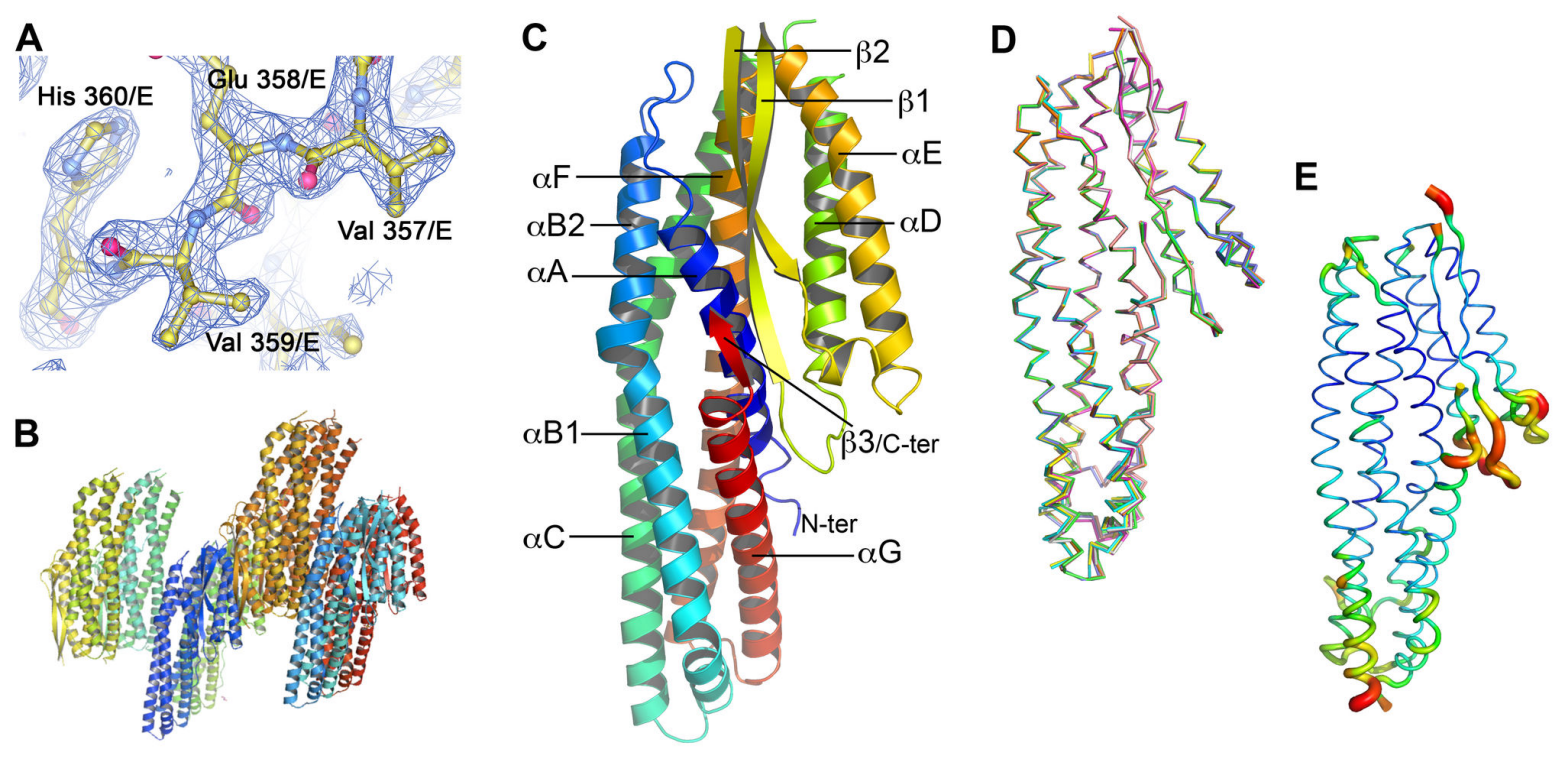
Overview of the structure of NheA. (**A**) Sample of electron density: the final (2F_obs_-F_calc_) exp(iα_calc_) map for the short C-terminal β-strand is contoured at 1σ and shown in blue; protein atoms are shown in ball and stick representation with yellow Carbon, blue Nitrogen, and red Oxygen atoms. (**B**) View of eight molecules in the asymmetric unit. (**C**) Cartoon representing the fold of NheA, rainbow coloured from blue at the N-terminal to red at the C-terminal, with secondary structures labelled. (**D**) Superposition of all eight molecules in the asymmetric unit of NheA, different monomers in different colours. (**E**) View of least well-ordered molecule (chain D) of NheA with regions of high temperature factor represented as wide orange/red tubes.

The NheA crystal structure shows that there are eight copies of the NheA molecule in the asymmetric unit (Figure 1B). There was no evidence of oligomerization as all eight molecules of NheA all appear to be monomeric using the criteria of PDBePISA [21]. Unless otherwise stated, all residues are numbered excluding the 26-residue N-terminal signal sequence [8,22], which is not present in the expressed protein.

The overall fold of each copy of NheA (Figure 1C) is well-defined but in all eight copies there are regions of disordered density at the N-terminal (between four and 18 residues undefined), in the loop connecting αC to αD (residues 168-171) and between β-strands 1 and 2 (217-222). In some copies there is also disorder at the beginning of helix αC (Figure 1E). Excluding these regions of disorder, the alpha carbons of the eight molecules superpose on one another with an RMSD of 0.3-0.4 Å and therefore can be considered as almost identical (Figure 1D). Consequently, unless otherwise stated, discussion will be limited to molecule A, which is the best defined.

As expected from the structures of Hbl-B [13] and ClyA [14], with which NheA has 20% and 18% sequence identity respectively, the structure of NheA is predominantly composed of α-helices with approximate dimensions of 95Å x 40Å x 20Å (Figure 1C). Like Hbl-B and ClyA, NheA can be regarded as consisting of two domains. The tail domain forms the main body of the structure and consists of five major helices designated: αA (Ser 24- Gln 44); αB (Leu 56 – Gly 104) with a distinct kink at residues TYR 95 and TYR 96; αC (Glu 110 - Leu 166) with a kink at residue Leu 141; αF (Ala 271- Glu 319); and αG (Ser 326 – Thr 355) (Figure 1C). The head domain includes two long α-helices: αD (Asp 172-Leu 195) and αE (Ser 243-Thr 269) separated by a β-hairpin consisting of two long β strands: strand 1 (Gly 204- Thr 216) and strand 2 (Thr 222- Leu 233). Unlike ClyA the two strands in this hairpin actually form part of a three-stranded β-sheet as Strand 1 forms hydrogen bonds to a third, very short β-strand from the tail domain, which is formed from 4 residues (Val 357, Glu 358, Val 359, His 360) at the C-terminal of the protein (Figure 1C).

### Related toxin structures

**Figure 2:**
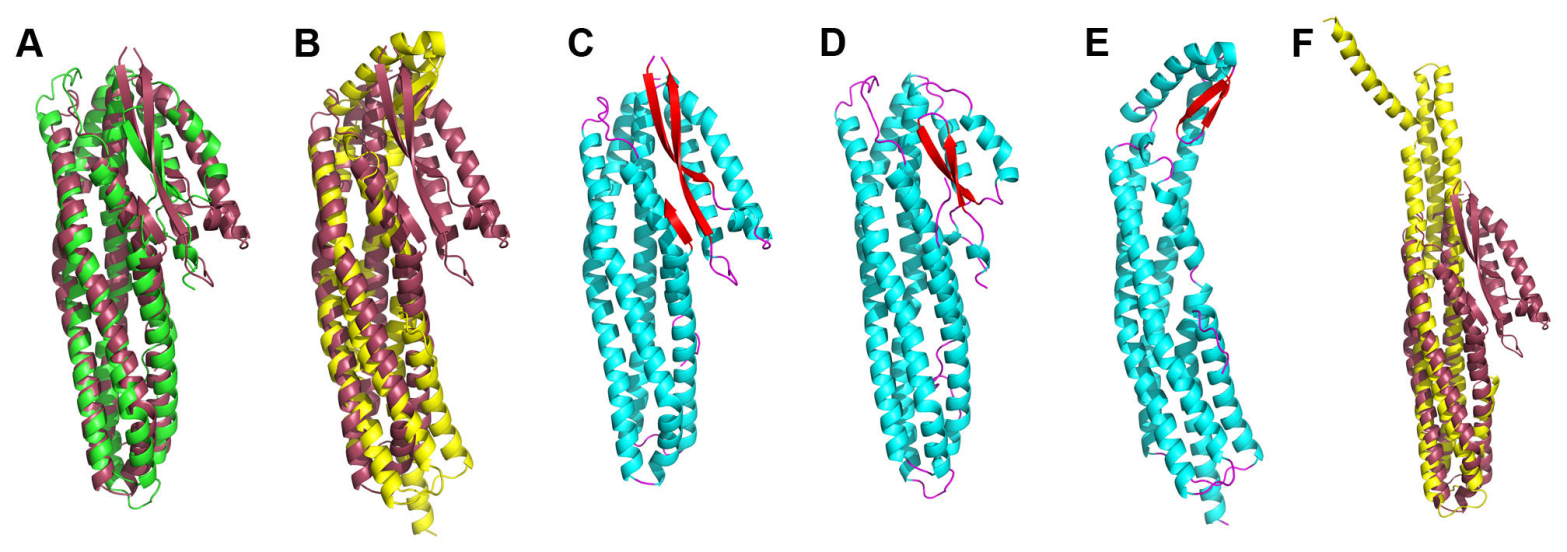
Comparisons of NheA with Hbl-B and the soluble form of ClyA. (**A**) Superposition of the structures of Hbl-B (green) and NheA (burgundy). (**B**) Superposition of the structures of the soluble form of ClyA (yellow) and NheA (burgundy). (**C**) Cartoon of NheA with α-helices coloured cyan, β-strands coloured red and loops coloured magenta. (**D**) Cartoon of Hbl-B coloured as in (C). (**E**) Cartoon of soluble form of ClyA coloured as in (C). (**F**) Superposition of the structures of a protomer from the pore form of ClyA (yellow) and NheA (burgundy).

A Dali search [23] was conducted and showed that the strongest structural similarities of NheA were with Hbl-B [13] (Figure 2A) and the soluble form of ClyA [14] (Figure 2B) although parts of the tail domain also superpose with the equivalent parts of the pore form of ClyA [18] (Figure 2F). This is consistent with the 20% and 18% sequence identities to Hbl-B and ClyA, respectively, reported by Fagerlund et al [24]. The resemblances detected are mainly in the alpha helical bundles that form the tail domains of these proteins as the head domains differ considerably in size and orientation (Figure 2). The tail domains of NheA, Hbl-B and the soluble form of ClyA are similar, consisting of a bundle of 5 α-helices with the same unusual topology reported for ClyA [14], although αA is significantly shorter in NheA than in the other two toxins. The head domain and β-sheets in NheA are longer than those in either Hbl-B and ClyA, but are nevertheless similar in overall fold: all three proteins have head domains composed of two β-sheets and two α-helices, all with the unusual topological arrangement first seen in ClyA [14]. Hbl-B and ClyA differ in the orientation of the head domain relative to the tail domain [13] and NheA resembles Hbl-B in this respect (Figure 2), and in both there is an interaction between the β-hairpin and the C-terminus of the protein. Thus in NheA and Hbl-B, the head domain is pointed downward and thus makes interactions with its tail domain, whereas in ClyA the head domain is turned upward making minimal interactions with the tail domain. In spite of this variation between different toxins, there appears to be no significant variation in the orientation of the head domain with respect to the main body of the molecule among the eight copies of NheA in the asymmetric unit (Figure 1D).

## Discussion

### Comparison with ClyA

ClyA is the best understood member of the ClyA/Hbl/Nhe superfamily of toxins. Indeed, it is probably structurally the best characterized of any α-pore-forming toxin, being known in both its soluble [14] and pore [18] forms. Each ClyA monomer is known to undergo extensive structural changes to form a protomer in a 12-meric α-helical transmembrane pore [18]. The Dali superposition of NheA on the soluble form of ClyA is shown in Figure 2B, and the two structures are shown individually in Figure 2C and 2E. In the NheA structure the N-terminal helix (αA) is much shorter than in soluble ClyA [14] where it reaches down the whole length of the molecule and briefly forms a full part of the 5-helix bundle (αA, αB, αC, αF, and αG) that occupies the lower half of the tail domain. This shorter αA in NheA may be a significant difference as a large movement of αA in ClyA is a key part in the transformation of the latter into its pore form [18].

### Relationship to Hbl toxins and the role of the β-tongue

**Figure 3:**
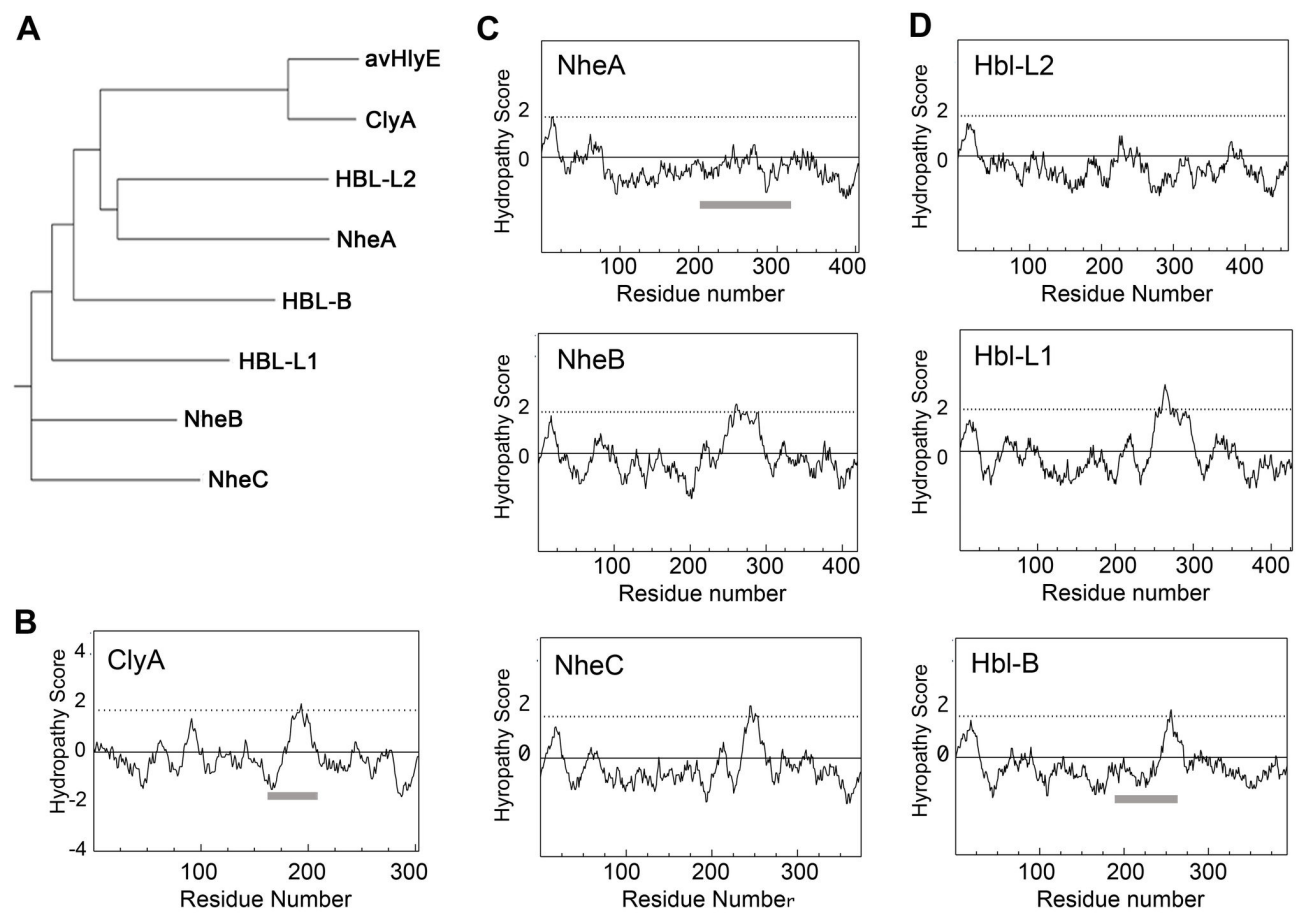
Sequence analyses of members of the Nhe, Hbl and ClyA superfamily. (**A**) Phylogenetic tree relating ClyA, avian HlyE (avHlyE), and the Hbl and Nhe components. (**B**) Hydropathy plot of ClyA sequence. (**C**) Hydropathy plots of NheA, NheB and NheC sequences. (**D**) Hydropathy plots of Hbl-L2, Hbl-L1 and Hbl-B sequences. Hydropathy scores calculated using Kyte-Doolittle score [25], calculated with a window width of 19 residues and the vertical axis representing increasing hydrophobicity. The dotted lines at 1.8 units represent the threshold for probable transmembrane sequences, horizontal grey bars indicate the extents of the head domain regions in ClyA, Hbl-B and NheA. Residue numbers include the 26 residue and 9 residue signal regions present in the sequences of NheA and Hbl-B, respectively, but not present in the expressed proteins.

Examination of a phylogenetic tree relating the three Nhe toxins and the three Hbl toxins together with two ClyA sequences from different strains of *E. coli* (Figure 3A) shows that NheA and Hbl-L2 are more closely related to one another than they are to the other Nhe and Hbl components. This is also reflected in their hydropathy plots [25] where it can be seen (Figure 3C, D) that NheA and Hbl-L2 both completely lack the hydrophobic sequence in their head domain regions that is crucial in ClyA function (Figure 3B). The hydrophobic sequences found in NheB and Hbl-L1 are much longer (~60 residues) than those found in NheC, Hbl-B and ClyA (~30 residues), and it could be speculated that the NheB and Hbl-L1 sequences could in principle be sufficiently long to form two transmembrane helices that could cross the membrane and return again to the same side.

While ClyA is a homo-oligomeric PFT, inducing hemolytic activity on its own, both NheA and Hbl-B require their companion proteins (NheB and NheC, and Hbl-L1 and Hbl-L2, respectively) for toxicity. Thus, it is predicted that the L1 and L2 components of Hbl toxins are required to stabilize the head domain of Hbl-B in membrane insertion or for inducing conformational changes in Hbl-B [13], whereas NheA has been shown to be mandatory as the last binding step to the Nhe complex, leading to cell lysis [10].

### The importance of NheA in the Nhe complex

The predicted β-tongues of NheB and NheC appear to have a major role in binding to cell surfaces, while in NheA it is seen to be amphipathic and is longer than those predicted in NheB and NheC [10]. The non-ionic detergent, dodecyl maltoside (DDM), acts as a membrane mimic component for ClyA [14,18,26]. Recent findings showed that pre-incubation of NheB with DDM resulted in a large molecular weight complex and prevented the binding of the protein to Vero cell monolayers, but this was not observed for NheA [27]. 1-anilinonaphthalene-8-sulphonic acid (ANS) fluorescence studies, monitoring the changes in fluorescence as a marker for protein conformational changes where exposure of hydrophobic regions of proteins favor increased binding and fluorescence [28], indicate that NheB exposed to DDM leads to changes in ANS fluorescence, whereas no ANS fluorescence changes were observed for NheA with DDM [27]. This possibly indicates that there are no conformational changes in NheA when exposed to DDM. This is supported by the fact that when NheA was treated with DDM no molecular weight shift was observed during the size exclusion chromatography analysis [27]. Also, DDM-treated NheA showed no increase in mass during differential dialysis experiments, indicated by the fact that the protein was able to pass through a 50-kDa cut-off membrane after exposure to DDM (Phung and Hardy, unpublished data). The αF and αB helices of NheA have one tryptophan residue each and a tryptophan scan of NheA would indicate the location of the conformational changes, if any, when the protein is exposed to DDM. But an NheA tryptophan scan shows that there is no movement of these residues upon addition to DDM (Phung and Hardy, unpublished data), indicating that the protein does not undergo any conformational changes in this region of the structure, in agreement with the ANS fluorescence studies on NheA [27]. These findings support the model in which conformational changes and oligomerization of NheB is a prerequisite event in the pore formation process which is completed by the insertion of NheA.

### Role of the β-tongue in NheA

The above discussion begs an important question: how does NheA carry out the crucial final step of pore formation? In ClyA the hydrophobic β-tongue undergoes a major conformational change to become part of a hydrophobic transmembrane helix that crosses the membrane; additionally the inside of the ClyA pore is lined by the amphipathic N-terminal α-helix which undergoes a large-scale movement to occupy its new position [18]. It seems unlikely that NheA (and by implication Hbl-B in the Hbl toxin) can do this - this is because the structure presented here shows that, although there is a β-tongue structure in NheA, its sequence alternates between hydrophobic and hydrophilic residues. The β-tongue therefore cannot plausibly form either a hydrophobic helix or an amphipathic helix capable of traversing the membrane. Moreover the second factor in ClyA pore formation, the N-terminal helix (αA), in NheA is much shorter than in ClyA which also suggests it cannot form a transmembrane pore in the same manner as ClyA [18].

**Figure 4:**
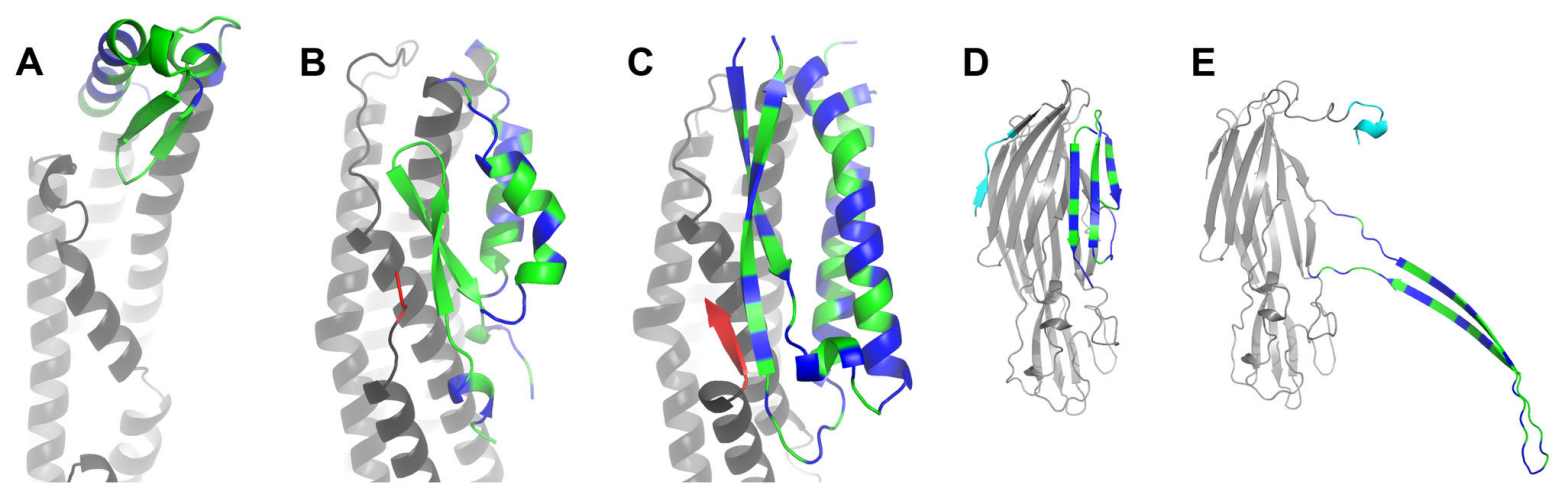
Cartoons of the head domains of Nhe, Hbl and ClyA and of *S. aureus* LukF and α-hemolysin. (**A**) The head domain of ClyA with hydrophilic residues coloured in blue, hydrophobic ones in green, and the tail domain in grey. (**B**) Head domain of Hbl-B coloured as (A) but with C-terminal coloured in red. (**C**) Head domain of NheA coloured as (A) but with C-terminal β-strand coloured red. (**D**) *S. aureus* LukF with the β-strands that form part of the transmembrane pore coloured as in (A); the remainder of the structure is coloured in grey, except for the N-terminal region which is coloured in cyan. (**E**) Structure of one protomer from the *S. aureus* α-hemolysin heptameric pore, coloured as in (D).

One intriguing possibility is that, rather than forming an α-helical pore like ClyA, NheA may, after the formation of an NheB prepore, form a transmembrane β-pore in a manner analogous to *Staphylococcus aureus* α-hemolysin [29]. The *B. cereus* toxin CytK belongs to the same β-barrel toxin family as the *S. aureus* α-hemolysin, which is an all β-sheet molecule completely unrelated to NheA, Hbl-B and ClyA. *S. aureus* α-hemolysin possesses a β-hairpin structure (Figure 4E) that spans the membrane as part of a 14-stranded antiparallel β-barrel, which is created when it oligomerizes into a heptameric structure. Because of the alternating hydrophilic and hydrophobic amino acids in this region, this sequence produces an amphipathic beta structure where the hydrophobic amino acids pack against the membrane and the hydrophilic amino acids form the water-facing part of the pore. The crystal structure of the soluble form of *S. aureus* α-hemolysin is not known, but that of a homologue, *Staphylococcal* leukocidin F (LukF), is available [30]. In LukF, this region of the structure also has a sequence of alternating hydrophobic and hydrophilic residues and is arranged into a 3-stranded sheet that has a hydrophic face and a hydrophilic face. The hydrophilic face is on the surface of the molecule while the hydrophobic face is packed against the main body of the molecule. Song et al. [29] have suggested that in the soluble form the β-strands of α-hemolysin are held in place by a "latch" that consists of a short N-terminal β-strand (Figure 4E, cyan), although this is not evident in the LukF structure [30] (Figure 4D, cyan). On α-hemolysin pore formation it has been proposed that the hydrogen bonds that secure the latch are broken and the hairpin is able to "swing out" so that its hydrophobic face interacts with the lipid and the hydrophilic face forms the inside of the pore as part of the heptameric pore assembly (Figure 4E). Intriguingly, we show here that in NheA a β-hairpin of similar sequence appears to be held in position by five hydrogen bonds from a short third β-strand that forms the C-terminal of NheA (Figure 4C, red). It is therefore tempting to speculate that the β-hairpin in NheA may also be capable of repositioning itself in order to form a transmembrane β- pore in a similar manner to *S. aureus* α-hemolysin.

There are a number of factors that need to be considered in conjunction with this proposal. The pore in the fully assembled Nhe toxin (NheA, NheB and NheC) is large, with an estimated diameter of 50 Å [11]. This is significantly larger than the *S. aureus* α-hemolysin pore which is about 14 Å in internal diameter at its narrowest point [29], and 24 Å on average [31], indicating that Nhe is unlikely to be a heptameric 14-stranded β-barrel like the former. This is not surprising since the optimal ratio between the Nhe components is about 10:10:1 (NheA:NheB:NheC), and one would then expect at least 21 monomers to be involved. Larger β-barrel pores with a higher state of oligomerization are possible; thus at the present time the largest known transmembrane β-barrels are the 22-stranded barrels in FhuA [32] and FepA [33] which both have diameters of approximately 40 Å [31]. In addition, the length of the β-strands must be sufficient to cross the membrane. In *S. aureus* α-hemolysin each strand consists of 13 amino acids, although the whole hairpin extends over 45 amino acids including the loops before between and after the strands (Figure 4E, lower right). In NheA the strands are 13 and 11 residues long, which is slightly shorter, but this could be augmented by residues from the loops and helices αD and αE. A more serious problem, however, concerns the types of hydrophobic amino acids in the proposed transmembrane β-strand. The most common amino acids for interacting with lipid tail groups in transmembrane proteins are phenylalanine, tyrosine, tryptophan, valine and leucine, while tyrosine and tryptophan are particularly abundant in the interface region between the inner and outer parts of the bilayer [34,35]. The hydrophobic residues in the NheA β-tongue, however, do not include tyrosine or tryptophan and moreover would contain a number of outward facing non-hydrophobic amino acids if the more hydrophobic side of the β-tongue faced outwards in a putative transmembrane β-barrel. This suggests that if the NheA hairpin did form a multimeric barrel structure, it would not interact directly with the membrane, but rather with NheB through the hydrophobic residues from a prepore formed by the proposed NheB transmembrane helices (see above). The third component, NheC, is present in low quantities but is essential for pore formation [8,10], perhaps using a similar mechanism for intial insertion into the membrane to that proposed for ClyA [18]. The specific function of NheC during the formation is still unknown, although it can be speculated that its role is catalytic and that NheC may not be a part of the pore. This is supported by the findings of Lindbäck et al [10] showing that the concentration of NheC can be reduced by 100 fold and cytotoxicity is still observed.

In conclusion, the structure of NheA has revealed an architecture showing it to be, like Hbl-B, a member of the ClyA toxin superfamily of α-pore forming toxins (α-PFTs). Nevertheless, in spite of this clear family resemblance it does not possess the hydrophobic β-tongue structure that is characteristic for Hbl-B and ClyA. Instead it has an amphipathic β-tongue that is secured by a short β-strand latch to the main body of the protein. Extraordinarily in this respect it is reminiscent of the archetypal β-sheet pore forming toxin LukF/α-hemolysin of *S. aureus*, suggesting that there may be a relationship in the mode of action between these apparently diametrically opposed classes of pore forming toxins.

It is clear that, in order to better understand the mechanism of the pore formation of Nhe, further functional and structural studies of the Nhe complex are required, with particular reference to the role of the β-tongue in NheA.

## Materials and Methods

### Gene cloning, expression, and protein purification

The target gene for NheA was cloned from *B. cereus* NVH 75/95 genomic DNA using primers NVH1339‑F 5′-GTGAAAAAGACTTTAATTACAGG- 3′ (forward) and NVH1340-R 5′ TTAATGTACTTCAACGTTTGTAA-3′ (reverse) in a pEXP5-CT/TOPO (Invitrogen) vector. Expression and purification of NheA was performed as previously described [36]. Selenomethionine (SeMet)-substituted NheA was expressed in *E. coli* strain BL21-DE3 in the SeMet-supplemented minimal medium with amino acids L-Isoleucine (50 mg/L), L-Leucine (50 mg/L), L-Lysine (100 mg/L), L–Phenylalanine (100 mg/L), L –Threonine (100 mg/L) and L–Valine (50 mg/L), together with L-SeMet (60 mg/L) and bases such as Adenine (0.50 g/l), Guanine (0.50 g/l), Thymine (0.50 g/l), Uracil (0.50 g/l). IPTG (200 mg/L) was added to induce expression of SeMet-incorporated NheA. The media also included MgSO_4_.7H_2_O (0.01 mg/ml) and Thiamine (0.004 mg/ml). The level of expression was in the range of 5-7% of total cell soluble protein. The purification of the selenomethionine-labeled protein was essentially the same as for native NheA [36], although some modifications were made to the procedure. Four different chromatographic steps were used: a DEAE sepharose fast flow ion exchange column was used as the initial step; the eluted SeMet- NheA fractions were cut using 2 M-2.5 M of (NH_4_)_2_SO_4_ for 2 hours at 4°C; then gel filtration and hydroxylapatite chromatography were applied in the reverse order compared to native protein purification; and finally an additional anion exchange chromatography step on a Resource Q column HPLC was used as a polishing step to remove small contaminant proteins. The protein was purified to at least 90% purity, as estimated by SDS-PAGE. The yield was ca. 3 mg from a 1 liter culture. Both Native and SeMet-substituted NheA were analyzed using liquid chromatography-mass spectrometry (LC-MS). The amino acid sequence of NheA shows the existence of seven methionine residues. Theoretically, when replacing methionine by selenomethionine the molecular weight of NheA would increase by approximately 47 Dalton per selenomethionine. Mass spectrum analysis of SeMet-NheA confirmed that all seven methionines were ~100% replaced by selenomethionines.

### Crystallization, data collection, and structure determination

The native NheA was crystallized using the hanging drop vapor diffusion method as described previously [36]. The native NheA crystals belong to monoclinic space group *C2*, with a calculated Matthews coefficient [37] of 2.21 Å^3^/Da, assuming 8 molecule/asymmetric unit (see Table 1). These crystals were flash frozen by immersion in liquid nitrogen following addition of 25% (v/v) ethyleneglycol to the mother liquor. X-ray diffraction data were collected from crystals flash frozen in a steam of nitrogen gas at 100 K using an Oxford Cryosystems Cryostream device. 25% ethyleneglycol (Hampton Research) was used as a cryoprotectant. X-ray diffraction data were collected on the I03 beamline at the Diamond Light Source in Oxford, United Kingdom. Full details of the native data processing have been previously reported [36] but are summarized in Table 1. Crystals of SeMet-NheA were obtained after 5 days from a condition containing 0.2 M (NH_4_)_2_SO_4_, 0.1 M Bis-Tris pH at 6, and 27 % PEG 3350. MAD data collection on the SeMet-substituted was performed at beamline I02 at the Diamond Light Source in Oxford, United Kingdom using an ADSC Q315 CCD detector, with the detector distance set at 225.0 mm. An exposure time of 0.75 second was used for each image and, data were collected at three wavelengths, (peak, inflection and remote). The space group of SeMet-NheA was *C2* and cell dimensions were *a* = 307.9 Å, *b*= 58.7 Å, *c*= 172.7 Å α=90°, β=110.4°, γ =90°, very similar to those of the native crystals (Table 1). The data collection and processing statistics are listed in Table 1. Previous studies on the native crystals [36] had indicated on the basis of Vm values [37] and from consideration of evidence of pseudo-translational symmetry, that there were probably eight molecules in the asymmetric unit. Each NheA molecule contains seven methionine residues and consequently it was expected that 56 SeMet would be present in the asymmetric unit. 50 of the 56 possible selenium atom positions were located using the SHELXC/D programs [38]. PHENIX [39] was then used for phasing and density modification and for automated model building. Eight chains were built and 2754 residues (95.6% of those present in the crystal) were located without manual intervention, although some of these were later removed during rebuilding. The polypeptide chain was built manually using the program COOT [40]. Refinement of the model was performed using the program REFMAC [41]. Final refinement statistics are given in Table 2: the final model contains 2674 residues (92.8% of those present in the crystal) and 1507 water molecules; the R_work_ and R_free_ were 0.21 and 0.26, respectively, at a resolution of 2.05 Å. Most of the residues in the eight molecules of NheA are well ordered, with the exception of N terminal and turn regions in all eight monomers of NheA, where the experimental electron density was poorly defined. PROCHECK [42] indicated that the refined model was in a good agreement with expectations for structure within this resolution range. The electron density map is of high quality (Figure 1A). All molecular figures were prepared with PyMol (www.pymol.org), unless otherwise stated. Contacts were analyzed using PISA http://pdbe.org/PISA [21]. The PROMOTIF program was used to analyze secondary structures in the NheA protein (available at http://www.ebi.ac.uk/pdbsum/) [42,43] and geometry was analyzed with MolProbity (available at http://molprobity.biochem.duke.edu/) [44] with a MolProbity score of 1.43, which places the model in the 98th percentile for structures with a resolution of 2.05 Å ± 0.25 Å.

**Table 1:** Data collection statistics.

**Data collection statistics**	**Native NheA**	**SeMet NheA**		
Space group	C2	C2		
Cell dimensions				
*a*, *b*, *c* (Å)	308.7, 58.2, 172.9	307.9, 58.7, 172.7		
α, β, γ (°)	90, 110.5, 90	90, 110.4, 90		
		**Peak**	**Inflection**	**Remote**
Beamline / Detector	Diamond I03	Diamond I02	Diamond I02	Diamond I02
Wavelength (Å)	0.9686	0.9794	0.9797	0.9784
Resolution (Å)^a^	50.2-2.05 (2.16-2.05)	29.8-2.05 (2.16-2.05)	29.9-2.09 (2.21-2.09)	29.9-2.19 (2.31-2.19)
No. of observations ^a^	504907 (74251)	672961 (96285)	629433 (89066)	547829 (76626)
No. of unique reflections ^a^	179016 (26237)	182607 (26191)	170815 (24262)	148979 (21012)
*R*_*merge*_^a,b^	0.077 (0.358)	0.114 (0.626)	0.10 (0.641)	0.106 (0.656)
*I / σI* ^a^	7.5 (2.5)	7 (2)	7.7 (2)	8.3 (2)
Completeness (%)^a^	98.9 (99.7)	99.6 (98.5)	99.5 (97.6)	99.3 (96.5)
Multiplicity ^a^	2.8 (2.8)	3.7 (3.7)	3.7 (3.7)	3.7 (3.6)
Number of molecules in the asymmetric unit	8			
Solvent content (%)	44.3			
Matthews coefficients (Å3/Da)	2.21			

Data collection statistics for the native NheA crystal are reprinted from [36] under a CC BY license, with permission from IUcr (International Union of Crystallography), original copyright 2012.

a values for the outermost resolution shell are given in parentheses.

b *R*_*merge*_ *= Σ|I - <I>|/ΣI*, where I is the integrated intensity of a given reflection.

**Table 2:** Refinement statistics.

**Refinement statistics**	**Native NheA**
Resolution (Å)	2.05
No. Reflections (working/free sets)	169739/8958
*R*_*work*_ * / R*_*free*_^a,b^	0.2118/0.2616
No. Atoms	22683
No. Residues	2674
No. Waters	1507
Average B values (Å^2^)	
Protein (all/main chain/side chain)	32.2/29.4/35.0
Water	37.1
RMS deviations from ideality	
Bond lengths (Å)	0.006
Bond angles (°)	0.876
Ramachandran plot	
Most favored regions (%)	95.9
Additionally allowed regions (%)	3.6
Generously allowed regions (%)	0.4
Disallowed regions (%)	0

a *R*_*work*_ *= Σ||F*(*obs*) | *|F*(*calc*) *||/Σ|F*(*obs*) | for the 95% of the reflection data used in refinement

b *R*_*free*_ *= Σ||F*(*obs*) | *|F*(*calc*) *||/Σ|F*(*obs*) | for the remaining 5% of the reflection data excluded from the refinement

### Accession numbers

Coordinates and structure factors for the NheA structure have been deposited at the Protein Data Bank with accession code 4K1P.
